# Dynamics of organizational climate and job satisfaction in healthcare service practice and research: a protocol for a systematic review

**DOI:** 10.3389/fpsyg.2023.1186567

**Published:** 2023-07-13

**Authors:** Silvina Santana, Cristina Pérez-Rico

**Affiliations:** ^1^Department of Economics, Management, Industrial Engineering and Tourism, University of Aveiro, Aveiro, Portugal; ^2^Research Unit on Governance, Competitiveness and Public Policies, University of Aveiro, Aveiro, Portugal; ^3^Economía de la Empresa Department, Rey Juan Carlos University, Madrid, Spain

**Keywords:** organizational climate, job satisfaction, systematic review, protocol, healthcare services, PICCO, health care professionals, health care organizations

## Abstract

Organizational climate and job satisfaction have been established as fundamental pillars of research and practice in organizational behavior and organizational psychology, inspiring many explanations and operationalizations over time. In most sectors, global trends such as labor shortages, high rates of turnover and absenteeism, the need to increase productivity, and the interest in new work models concur to keep climate and job satisfaction on top of the research agenda. The situation is particularly acute in the healthcare sector, where related factors have the capacity to influence all aspects of care provision, including patient safety and the physical and mental health of care providers. Nevertheless, a gap in knowledge persists regarding climate, job satisfaction, and their relationships in healthcare services. This protocol describes a study that aims to examine the dynamics of climate and job satisfaction in healthcare organizations from the practice and research perspectives. The protocol complies with PRISMA-P. PRISMA will be used to report the results of the study. Databases will be searched for published studies in May 2023, and we expect to complete the study by December 2024. A framework based on a multi-dimensional concept of quality in research will be used to examine the quality of any studies before inclusion. The results will be disseminated in two systematic reviews. We will describe proposed models depicting the dynamics of climate and job satisfaction in healthcare organizations. We will systematize and discuss available evidence regarding the outcomes of climate and job satisfaction in healthcare work environments. We will synthesize information on research designs and methodological options of included studies. We will identify measures of climate and job satisfaction used in healthcare settings, assess their psychometric properties, and appraise the overall quality of underlying studies. Finally, we expect to identify areas in need of further research.

## 1. Introduction

### 1.1. Overview of climate research origins and evolution

Organizational climate, widely understood as employees' shared perceptions of organizational events, practices, and procedures (Patterson et al., 2005; James et al., 2008; Schneider et al., 2013; Pomirleanu et al., 2022), is central to organizational behavior theory and practice. As a variable positioned between the context of an organization and the behavior of its members, the organizational climate would provide a way to understand how employees experience their organizations (James et al., 2008; Ryu et al., 2020).

The original study by Lawrence R. James (1943–2014) and colleagues distinguishes between psychological climate and organizational climate (Sleutel, 2000; Parker et al., 2003; James et al., 2008). The fact is, while current research is more focused on aggregate rather than on psychological climate (Schneider et al., 2000; Patterson et al., 2005; D'Amato, 2023), perceptions are formed at the individual level, where work environments are cognitively apprehended, represented, and appraised, in terms of their meaning to and importance for individual employees in organizations (James and Jones, 1974; James and Sells, 1981). The meanings imputed to work environmental attributes would be “phenomenological experiences, which is to say that they are cognitive constructions designed to interpret information sensed from the work environment,” while comparing to previously stored mental representations or schemas.

A bias toward the descriptive meaning of work environmental attributes was common in research work in cognitive science and experimental social psychology. However, the fact that measures of situational antecedents in climate research involve perception by individuals would bring forward an issue of individual assessment, not intrinsic to the definition of a particular variable, driving the research agenda toward “subjective interpretations of environmental attributes” (James et al., 2008, p. 9) and rising issues of evaluative and affective meaning, far beyond descriptive initiatives. As argued in a previous research (Patterson et al., 2004, p. 195), “Description and affect are (…) likely to be combined in responses to at least some climate items.”

Based on previous research identifying four latent factors representing the most important personal, work-related values (Locke, 1976), four distinct domains of roles, jobs, leaders, and work groups have been consistently proposed as first-order dimensions of psychological climate. As organizational research often involves multiple levels of analysis, a composition theory for climate has been proposed (James, 1982) and models available in the literature (Chan, 1998) were applied to this field (Dawson et al., 2008; Wallace et al., 2013; Zacher and Yang, 2016). It has been suggested (Schneider et al., 2011) that, absent from group-level assessment, one cannot conclude that measures of organizational climate truly reflect the properties of a group, making it irrelevant for practical improvements in an organization. Aggregation depends on agreement on measures of psychological climate perceptions, and different indices have been developed to measure interrater agreement (Cardona Echeverri and Zambrano Cruz, 2014; Hsiung et al., 2020; Powell et al., 2021).

The impact of organizational climate on individual outcomes (Gershon et al., 2007; Clarke et al., 2011; Thompson and Rose, 2011; von Treuer et al., 2014; Loh et al., 2019), organizational outcomes (Patterson et al., 2004, 2005; Berberoglu, 2018), and healthcare outcomes (MacDavitt et al., 2007; Roch et al., 2014) is well documented in the literature, either by using mean psychological climate scores or other statistical functions of psychological climate scores, such as climate strength (Schneider et al., 2002, 2013; Afsharian et al., 2018) and climate quality (Lindell and Brandt, 2000) as mediators of antecedents and outcomes. Many times, perceptions of dimensions of work or workplace environment are investigated as determinants of individual outcomes (Zhenjing et al., 2022), without a reference to organizational climate as a concept. Very frequently, the climate is investigated as an antecedent of burnout (Thompson and Rose, 2011; Junça-Silva and Freire, 2022) and physical and psychological problems of healthcare personnel (Gershon et al., 2007; Loh et al., 2019), with increase in the literature after the health emergency caused by COVID-19 (Penconek et al., 2021).

Overtime, the concept of organizational climate has been established as one of the pillars of organizational behavior and has inspired many explanations and operationalizations, yet with few well-achieved validations of measures of the construct (Patterson et al., 2005; Poghosyan et al., 2013). Moreover, in the healthcare sector, there are not many studies on the subject, and there is a gap in knowledge that it is important to analyze and fulfill with further research.

### 1.2. Job satisfaction in research and practice

Job satisfaction may be defined as “a pleasure or positive emotional state resulting from the appraisal of one's job or job experience” (Locke, 1976).

Five main theoretical approaches have been proposed to explain job satisfaction (Campbell et al., 1982), while the antecedents of job satisfaction have also been the object of competing theories (Baker, 2004). The range of affect theory (Locke, 1976) is perhaps the most famous job satisfaction model. The main assumption is that satisfaction is determined by a discrepancy between what one seeks in a job and what one gets from that job, and the value given to a specific facet of work would moderate the level of satisfaction achieved. The task characteristics approach (Hackman and Oldman, 2007) posits that task characteristics are related to employee attitudes, and recent studies seem to support a direct impact on job satisfaction (Bhuian and Menguc, 2002). The social information processing approach (Salancik and Pfeffer, 1978) holds that social cues processed from the work environment determine job attitudes; supervision and leadership styles or behaviors, for example, might have an impact on job satisfaction (de Vries et al., 1998). The dispositional theory defends that the individual possesses relatively stable unobservable mental states such as needs or attitudes that will impact their perceptions and behaviors (Staw and Cohen-Charash, 2005, p. 73). Traits would significantly influence their affective and behavioral reactions to organizational settings (Davis-Blake and Pfeffer, 1989), and individuals would process information in such a way to be consistent with their internal states. An integrated approach (Griffin et al., 1987) suggests that job enrichment and social cues combine to influence perceptions and attitudes.

Job satisfaction is usually understood as a global concept comprising various facets (Penconek et al., 2021; Tenaw et al., 2021), with research designs and measurement scales being proposed accordingly. Nevertheless, some argue that the so-called facets of job satisfaction are simply evaluations that individuals make about their work environment and that positioning job satisfaction as the result of an evaluation mediated by affect and beliefs calls into question the facet vs. global satisfaction distinction (Rafferty and Griffin, 2009).

Categorizations of job satisfaction outcomes include work-related aspects, labor market experiences, physical and mental health, social costs, and other attitudes. The turnover rate has been the most consistent measure to be associated with job satisfaction (Campbell et al., 1982; Mertala et al., 2022), but the relationship seems to be moderated by turnover intention, defined as conscious willfulness to seek other alternative job opportunities in other organizations (Campbell et al., 1982; Lambert et al., 2001). A great number of studies have also suggested a relationship between job satisfaction and the health of workers, a link confirmed by a meta-analysis of 485 studies with a combined sample size of 267.995 individuals (Faragher et al., 2005).

Research designs addressing job satisfaction range from qualitative and quasi-experimental field studies in early research to more recent studies using experience sampling methodologies to deal with intraindividual processes, while emphasizing the role of affect in job satisfaction and that affective reactions are unstable, yet the most common methodologies involve the use of questionnaires, with a diversity of measures available, including graphic measures (Rafferty and Griffin, 2009). A new domain of possibilities regarding the role and the importance of job satisfaction opens with studies incorporating job satisfaction in multilevel processes involving teams and organizations. In this line, previous research showed that aggregate job satisfaction mediated the link between a company's organizational climate and objective measures of company performance (Patterson et al., 2004).

Some argue that there is little consistency in the use of job satisfaction measures (Rafferty and Griffin, 2009). In the health sector, a systematic review (van Saane et al., 2003) aiming to select job satisfaction instruments of adequate reliability and validity to be used as assessment tools in hospital environments has found 11 work factors that might form the basis of the job satisfaction concept; only seven of the analyzed instruments met the reliability and validity criteria defined, and only four addressed job satisfaction of health professionals.

Concerning data analysis procedures, it has been highlighted that most empirical studies addressing job satisfaction, especially in healthcare settings, rely on descriptive statistics, analysis of variance, correlation, or regression analysis, with few attempts at using comprehensive models supported by a simultaneous test of hypotheses (Santana and Loureiro, 2019).

In the healthcare sector, global trends concur to keep job satisfaction as a hot topic on the research agenda. The escalation in healthcare costs calls for interventions aiming at increasing productivity and overall performance, aspects that have been connected to job satisfaction, both directly and indirectly (Kontodimopoulos et al., 2009; Pillay, 2009). High turnover rates, presenteeism and absenteeism (Rantanen and Tuominen, 2011; Belita et al., 2013), and persistent professional shortages increase concerns regarding recruitment, training, and retention of specialized staff, both in developed and developing countries (Fang, 2001; Bodur, 2002; Taunton et al., 2004; Lu et al., 2005; Arab et al., 2007; Coomber and Louise Barriball, 2007; Qian and Lim, 2008; Pillay, 2009; Shi et al., 2023). Patient safety (Haas et al., 2000; Rathert and May, 2007), patient satisfaction (Haas et al., 2000), and the total quality of health services (Bodur, 2002) may be jeopardized by dissatisfied staff (Pillay, 2009).

Nevertheless, several limitations have also been reported by systematic reviews regarding research on job satisfaction in healthcare organizations, including the inconsistent use of concepts and terminology, the heterogeneity of measures used and the quality of related studies (van Saane et al., 2003; Amiresmaili and Moosazadeh, 2013; Chen et al., 2022), and the lack of studies addressing specific health settings such as long-term care (Lee et al., 2020).

### 1.3. Bridging organizational climate and job satisfaction

Adding to the discussion on the relationship between psychological climate and affective response, three alternative models of the causal relationship between psychological climate and job satisfaction were proposed and tested (James and Tetrick, 1986; James et al., 2008). Subsequent studies (Mathieu et al., 1993) provided further support for a reciprocal model, where psychological climate appears to mediate the relationship between the work environment and affective reactions to that environment. Individuals' valuations of the work environment would elicit affective responses, and those affective responses would then influence the individuals' valuations of the environment, in the light of their beliefs and expectations. Other studies showed that there is substantial empirical overlap between some aspects of organizational climate and job satisfaction but concluded that organizational climate and job satisfaction are distinct concepts, even if “evaluative judgments cannot always be excluded from the measurement of climate” (Patterson et al., 2004, p. 213).

Recent empirical research investigated organizational climate as a determinant of job satisfaction (Tsai, 2014; Gaunya, 2016; Ahmad et al., 2017; Vidak et al., 2023), using a variety of models, scales, and procedures. Frequently, perceptions of dimensions of work or workplace environment are investigated as antecedents of job satisfaction, without a formal reference to organizational climate as a concept (Raziq and Maulabakhsh, 2015; Santana and Loureiro, 2019; Molina-Hernández et al., 2021), even if dimensions align with facets of psychological climate. Job satisfaction has been studied as a direct outcome of organizational climate (Lu et al., 2005) and also as a mediator between climate and other outcomes, such as productivity (Patterson et al., 2004) and turnover intention (Li et al., 2020).

Job satisfaction is a recurrent theme in the research literature in the fields of management sciences and health sciences because of its links with turnover, workers' health and wellbeing, patients' safety and satisfaction, and other practice-related outcomes. The results from the searches conducted with the working query in different databases during the preparatory phase of this study confirm this statement, as well as the little research available on organizational climate conducted in healthcare settings (see Appendix). A few systematic reviews dealing with organizational climate or job satisfaction in healthcare practice were found in the literature, but there was none dealing with both concepts and the relationships between them. Therefore, a systematic review was deemed necessary to examine aspects of climate and job satisfaction in healthcare organizations.

Given the inconsistencies in terminology and the lack of clear theoretical positioning found in many studies analyzed in the preparatory phase, we will consider studies addressing psychological climate and organizational (aggregate) climate, as defined by others (James et al., 2008), whenever conducted in healthcare organizations. For the sake of rigor and to avoid ambiguity, from now on, we will use climate to represent both concepts in framing discourse, always guaranteeing the use of the terminology proposed by a specific study when citing them.

### 1.4. Review questions

The objective of our study is to map the dynamics of organizational climate and job satisfaction in healthcare organizations from the practice and the research perspectives, by means of two complementary systematic reviews addressing the main research questions of the overall project: (1) To what extent and how have climate and job satisfaction been researched in organizations delivering healthcare services and what is the evidence on their impact? (2) How have climate and job satisfaction been measured in healthcare organizations and what is the quality of the evidence available in this regard?

Subsequently, we have defined the following specific questions (SQ) for review 1 and review 2.

#### 1.4.1. Review 1

R1SQ1: To what extent have the concepts of climate and job satisfaction been researched in healthcare organizations?

R1SQ2: What models have been proposed and tested by studies addressing climate and job satisfaction in healthcare organizations?

R1SQ3: What has been reported regarding outcomes of climate and job satisfaction in healthcare organizations and what is the quality of the evidence available in this regard?

R1SQ4: What practice-related challenges in dealing with climate and job satisfaction in healthcare organizations are reported in the literature?

R1SQ5: What characterizes the research designs and the methodological options of studies on climate and job satisfaction conducted in healthcare organizations?

R1SQ6: What challenges and future directions have been discussed regarding research on climate and job satisfaction in healthcare organizations?

#### 1.4.2. Review 2

R2SQ1: What measures of climate and job satisfaction have been used in healthcare organizations?

R2SQ2: What are the psychometric properties of measures of climate and job satisfaction used in healthcare organizations?

R2SQ3: What is the overall quality of studies on climate and job satisfaction in healthcare organizations?

R2SQ4: What challenges and future directions have been discussed regarding measures of climate and job satisfaction in healthcare organizations?

## 2. Methods

### 2.1. Study procedure

The study is following the best practice available, assuring rigor and replicability. The Cochrane protocol guide (Higgins et al., 2019) was used to guide the development of the study protocol. The protocol complies with Preferred Reporting Items for Systematic Reviews and Meta-Analysis Protocols (PRISMA-P, http://www.prisma-statement.org/documents/PRISMA-P-checklist.pdf). The Preferred Reporting Items for Systematic Reviews and Meta-Analysis (PRISMA, http://www.prisma-statement.org) (Liberati et al., 2009; Moher et al., 2009) will be used to report the systematic reviews. The systematic reviews have been submitted to the International Prospective Register of Systematic Reviews (PROSPERO) database and awaiting registration.

Review 1 and Review 2 address different specific research questions, by using the same primary studies to which the same eligibility criteria and screening process apply. We have established a participant/population, intervention, comparator, context, and outcome (PICCO) framework, a slightly modified version of the traditional PICO (participants/population, intervention, comparator, and outcome). A few limitations of PICO have been identified even in the health area (Huang et al., 2006). We expect it to underperform in situations that involve complex and fast-changing environments, over which observers, researchers, implementers, and users have little, or even no, overall control. Therefore, detailed description and characterization of context conditions and changes to context conditions are of paramount importance in understanding further developments and outcomes (O) of interventions (I) reflected on the participants/population (P), justifying the inclusion of a context (C) dimension in the framework. The PICCO framework will be used to develop and combine subject headings and keywords and to help analyze, synthesize, and report the results of both systematic reviews. The complementary systematic reviews will progress in the following way: formulation of research questions, literature search, selection of papers, extraction of data, appraisal of study quality, analysis of data, synthesis of data, and report and use of results. Extraction spreadsheets will be organized to serve Review 1 and Review 2, allowing data extraction and data synthesis to be performed concurrently for both reviews.

### 2.2. Eligibility criteria

The research will include studies fulfilling all the following requisites: (1) investigate climate and job satisfaction in organizations delivering health care services; (2) discuss measures and/or models used in research on climate and job satisfaction; (3) address individuals working in healthcare organizations in a position with a direct or indirect influence on delivering health care services, by the time they participated in a study on climate or job satisfaction in that healthcare setting. We will include studies conducted in public or private healthcare organizations independent of the country, such as hospitals, health centers and other primary care settings, units of integrated care networks providing health care services and other health care practices. Studies measuring only specific aspects of climate (e.g., autonomy) or job satisfaction (e.g., benefits) will be excluded.

The general outcomes defined for the study are climate and job satisfaction in healthcare organizations. Review 1 and Review 2 will consider studies that include the following specific outcomes, from the practice and the research perspectives. Review 1 main outcomes are: (1) effects/outcomes of climate and job satisfaction; (2) practice-related challenges in dealing with climate and job satisfaction in healthcare organizations; (3) models depicting dynamics of climate and job satisfaction in healthcare organizations; (4) research designs and methodological options of included studies; (5) challenges and future directions in research on climate and job satisfaction in healthcare organizations. Review 1 secondary outcomes are: (1) tested climate and job satisfaction measures; (2) psychometric properties of climate and job satisfaction measures. Review 2 main outcomes are: (1) tested climate and job satisfaction measures; (2) psychometric properties of climate and job satisfaction measures. Review 2 secondary outcomes are: (1) effects/outcomes of climate and job satisfaction; (2) practice-related challenges in dealing with climate and job satisfaction in healthcare organizations; (3) models depicting dynamics of climate and job satisfaction in healthcare organizations; (4) research designs and methodological options of included studies; (5) challenges and future directions in research on climate and job satisfaction in healthcare organizations.

Regarding study design, we will include all types of studies if other eligibility criteria are met. Namely, we will consider qualitative, quantitative and mixed methods research; longitudinal, cross-sectional, case control, cohort studies, and trials studies. Literature reviews and systematic reviews will be excluded but their references will be searched for relevant studies. Studies published in English, Portuguese and Spanish will be considered for inclusion. Studies published between January 2000 and the date of search will be included.

### 2.3. Search strategy

A three-step search strategy will be followed in this study. First, a limited search of SCOPUS and Web of Science databases will be undertaken followed by an analysis of text words contained in the title and the abstract of selected articles and of the keywords used to describe them. In the second step, a search using all relevant keywords and terms identified will be undertaken across all included databases. In the third step, the reference lists of all identified reviews and systematic reviews will be searched for additional pertinent studies. During the search process, several terminologies and spellings of the keywords will be considered as they may affect the identification of relevant studies. The following databases of published studies will be searched: SCOPUS, Web of Science, PUBMED, MEDLINE, and CINAHL.

### 2.4. Data management

Following the entry of the relevant keywords and identified terms in the search databases, the results will be exported into a folder and uploaded to Rayyan QCRI (Ouzzani et al., 2016). Rayyan will be used to eliminate duplicates and support the initial blind screening of the title and abstract and the blind assessment of full-text studies performed by the teams of reviewers.

### 2.5. Selection process

The selection process will evolve in three phases. In the first phase, the title and abstract of identified articles will be screened by two independent reviewers to decide on their advance to the second phase. Upon the completion of the title and abstract screening and resolution of any conflicts between pairs of reviewers, the retained studies will be assessed through full-text reading. The discussion will be used to resolve disagreements, and a third reviewer will be consulted if consensus cannot be reached. In the third phase, the studies included will be assigned to Review 1, Review 2, or both and marked accordingly. The process will be demonstrated using a PRISMA flow diagram for Review 1 and a PRISMA flow diagram for Review 2.

### 2.6. Data collection process and data items

Data will be extracted and collated by two independent reviewers onto predefined data extraction forms. The data extracted will include specific details about the population, intervention, context, outcome of significance to the primary and secondary questions, and aspects of research designs and methodological options of the included studies. Data extraction forms will be validated by the review team prior to utilization to ensure acceptability and study validity. Disagreements between the reviewers regarding extracted data will be resolved through discussion or with a third reviewer.

The following data and information will be extracted:

1. Structural characteristics of the study: author(s), title, affiliation of first author, type of publication, date of publication, and language.
2. Methodological characteristics of the study: the general aim of the study, theoretical/conceptual framework/tradition adopted, definitions provided of core concepts in the interventions, study research questions and/or hypothesis, sample size, other details on research design and methodological options (e.g., type of study and data analysis), and reported limitations.
3. Participants: professional category/provider role and other demographics.
4. Types of intervention(s)/phenomena of interest.
  Model: dimensions, antecedents/determinants, consequents/outcomes, mediators, and moderators.
  Measures: name of measure; domains, subdomains, the total number of items, method of administration, response options, and scoring.
5. Context: type of healthcare organization (e.g., hospital), study setting if applying (i.e., emergency room), and country.
6. Outcomes: reported effects/outcomes of climate and job satisfaction, validated models depicting dynamics of climate and job satisfaction in healthcare organizations, practice-related challenges in dealing with climate and job satisfaction in healthcare organizations, challenges and future directions in research on climate and job satisfaction in healthcare organizations, climate and job satisfaction measures, psychometric properties of climate and job satisfaction measures, and other measurement properties of interest.

### 2.7. Risk of bias/quality assessment

The methodological quality of included studies will be assessed by two independent reviewers. Any disagreements arising between the reviewers will be resolved through discussion and with a third reviewer, if necessary. The quality assessment will identify and exclude studies not meeting minimum research and/or professional standards (e.g., reporting mere opinions, suffering from ethical problems or some type of gross bias, and omitting sources of data) and will generate quality ratings to qualify the synthesis results. The psychometric properties of the measures used in each study will be assessed, as part of the quality assessment procedure, and tabulated to be used in the data synthesis in Review 2.

Considering the type of interventions sought and the expected complex nature of the contexts involved, it is anticipated that the likelihood of including studies based on experimental designs is low, that part of the included studies will be descriptive, and that those studies reporting on results from implementations will be based mostly on observational data and cross-sectional studies. We also expect high heterogeneity in study designs. Therefore, the direct applicability of well-reputed standardized tools for assessment of methodological validity of studies considered for inclusion in systematic reviews and meta-synthesis was uncertain, and a framework based on a multi-dimensional concept of quality in research will be used during the execution of the review to examine the quality of any studies under consideration. The framework covers relevance, conceptual depth and breadth, methodological rigor, and quality of reporting and will assess the description of the problem being addressed, conceptual soundness, the existence of definitions of central concepts, description of methodological approach, identification of the study objective(s), description of study/implementation context, identification of outcomes sought, description of the methods of data collection and analysis, evidence on selective reporting bias, choice of research design, evidence on quality of any data collected, the relevance of the results, and weight of evidence.

### 2.8. Data synthesis

Data extracted from the studies included will be summarized numerically, by describing the number and characteristics of studies in overview tables, and narratively, by synthesizing data around the defined concepts and data items. We will perform a realist synthesis - a narrative synthesis (Popay et al., 2006) with a focus on the relationship between context, mechanisms under research, and outcomes (CMO) (Pawson, 2002; Pawson et al., 2005; Rycroft-Malone et al., 2012) - to cross-analyze and synthesize the findings regarding the characteristics of populations, contexts, and interventions, the direction and size of effects, the various plausible mechanisms at work, and how they interact with context to determine outcomes. To deliver the answers to R2SQ2 and R2SQ3, the psychometric properties of the measures in each study will be graded, and the studies will be grouped by measure to produce a qualitative summary, the pooled ratings for all measurement properties, and, finally, the overall grade of the quality of evidence on each measure. All ratings will be performed independently by two reviewers.

The synthesis will be performed with a focus on concepts and themes (Popay et al., 2006), namely, those concepts and themes that would allow us to understand, or even explain, questions related to population-context-mechanism (activated by the intervention)-outcomes interactions. To summarize and report a synthesis across all the included studies, we will develop a provisional theory of how and why the intervention works (Popay et al., 2006) against which to compare the findings of the different studies in an iterative way. This will lead to increasingly complete versions of the preliminary synthesis until all the evidence in the available data is used.

We will consider the effect of any moderator variables (Popay et al., 2006) and will use the Weight of Evidence (WoE) rating (Gough, 2007) to examine the robustness of findings and to summarize the reviewers' assessment. We will evaluate the feasibility of a sensibility analysis after having a precise idea of the data available from the primary quantitative studies as we expect most studies to have a non-experimental nature.

The methods were chosen due to the foreseen nature of the studies to be included and our interest in understanding the contexts and the mechanisms that are part of the explanation of the outcomes of a given intervention, and because the realist synthesis will allow us to assess the external validity of the primary studies (van der Knaap et al., 2008).

Given the type of interventions sought and the diversity of the contexts involved, and considering the studies analyzed during the pilot phase, we ruled out the possibility of performing a standard meta-analysis. Still, graphic analyses will be considered after having a precise idea of the data available from the studies included.

At this stage of the review, there will be no further filtering of primary studies, involving the application of further exclusion criteria.

## 3. Results

The protocol here reported, by the nature of its theme, objectives, and methodology, lays down a valuable space and opportunity to address the dynamics of organizational climate and job satisfaction in healthcare services practice and research while delivering rigorous guidelines for evidence search, systematization, assessment, and reporting in this field.

We defined clear specific objectives for the study, identified the guidelines used to report the protocol and the upcoming systematic reviews, and described the PICCO framework to be used to develop and combine subject headings and keywords in the search phase and to help analyze, synthesize, and report the results of both systematic reviews.

We delivered eligibility criteria anchored on a PICCO framework and the required general characteristics of primary studies considered for inclusion in the research. We set up a search strategy designed to minimize bias and errors. We defined research methods that allow us to identify, categorize, analyze, and report aggregated evidence on the dynamics of organizational climate and job satisfaction in healthcare services practice and research while minimizing the risk of bias and errors in analysis and reporting. Following the best guidance and practice available, we previewed methods to assess and report the quality of the studies included.

Preliminary searches were conducted in February 2023. Databases will be queried for published studies on May 2023, and we expect to complete the review by December 2024.

## 4. Discussion

To the best of our knowledge, the protocol reported in this paper is unique in its objectives and methodology. Results from searches conducted with the work query in different databases during the preparatory phase of this study confirm this statement, as well as the little research available on organizational climate conducted in healthcare settings. A few systematic reviews dealing with organizational climate or job satisfaction in health care practice were found in the literature but, generally, those studies did not conform to relevant guidelines and were not built on clear methods, defined a priori, to identify, categorize, analyze and report aggregated evidence on a specific topic. Moreover, we could not find any published protocol for a systematic review or a complete systematic review dealing with both concepts and the relationships between them.

The study will deliver breadth and depth in mapping and assessment, including prevalence of research and their quality, measures of climate and job satisfaction and their psychometric properties, models and research methodologies, reported outcomes from climate and job satisfaction in healthcare organizations.

A major strength of our study is that it will provide evidence on dynamics of climate and job satisfaction in healthcare organizations published in the 21 century, while uncovering and assessing gaps in theoretical and applied research in these fields. A limitation of this protocol is that, while covering the languages spoken by a very significant part of the world population, it will not be able to rule out some cultural and geographic bias, due to the exclusion of studies published in other important languages, such as Mandarin Chinese, Hindi and Arabic.

## 5. Conclusion

Study protocols are instrumental in guaranteeing strict guidance, replicability, consistency, accountability, transparency and learning in evidence-based research and practice, regardless of disciplinary and application field. They represent the first step toward the construction of a rich, trustworthy, evidence-based knowledge base and act as references for fellow researchers, policy makers and other stakeholders. This is particularly true in the core area of our study, where a preliminary search of available literature has revealed a fragmented research field whereas there is an urgent need to promote awareness and investment in work conditions and job satisfaction of health care professionals.

The results from the application of this protocol will be reported in two systematic reviews, addressing different aspects of research on climate and job satisfaction and in part using different analysis and reporting techniques and tools. Yet, the pool of primary studies and the eligibility criteria are the same, therefore conditions are in place to research both conceptual aspects, outcomes in work settings and methodological aspects within the same universe of primary studies and time frame and integrate the results in meaningful way.

We anticipate that the results will help to contextualize and integrate disciplinary- and specific- outcomes-oriented approaches and facilitate the work and decision-making of health care professionals, managers in healthcare organizations, policy makers, governmental agencies, regulators, funders, advocacy groups, and researchers. Finally, we expect to identify areas in need of further research, especially in the intersection of disciplinary fields. The potential to advance available knowledge and contribute to better work practices is high, as many of the areas addressed by this work are underserved by published literature.

## Data availability statement

The original contributions presented in the study are included in the article/supplementary material, further inquiries can be directed to the corresponding author.

## Author contributions

SS conceptualized the study. SS and CP-R carried out the preliminary database searches, were involved in the selection and analysis of studies that form the bibliographic support of this protocol, piloted the study selection process, wrote and edited the original draft, and approved the final submission.

## Appendix

**Table A1:** Number of articles retrieved with the working query from different databases, during the preparatory phase.

**Database**	**Topic**	**Number of articles**
PubMed	Organizational climate or job Satisfaction in healthcare settings	163
	Organizational climate or job satisfaction in general	487
	Organizational climate in healthcare settings	13
	Job satisfaction in healthcare settings	150
	Organizational climate in general	26
	Job satisfaction in general	464
Medline	Organizational climate or job satisfaction in healthcare settings	2.329
	Organizational climate or job satisfaction in general	4.138
	Organizational climate in healthcare settings	475
	Job Satisfaction in healthcare settings	2.017
	Organizational climate in general	813
	Job satisfaction in general	3.493
Scopus	Organizational climate or job satisfaction in healthcare settings	2.714
	Organizational climate or job satisfaction in general	7.627
	Organizational climate in healthcare settings	144
	Job satisfaction in healthcare settings	2.607
	Organizational climate in general	513
	Job satisfaction in general	7.207
WoS	Organizational climate or job satisfaction in healthcare settings	2.185
	Organizational climate or job satisfaction in general	10.798
	Organizational climate in healthcare settings	189
	Job satisfaction in healthcare settings	2.026
	Organizational climate in general	862
	Job satisfaction in general	10.096
